# Supplementary material for: Sensitivity to Immune Checkpoint Blockade in Advanced Non-Small Cell Lung Cancer Patients with EGFR Exon 20 Insertion Mutations
Source: Genes (Basel). 2021 Apr 30;12(5):679. doi: 10.3390/genes12050679 (PMC8147255; doi:10.3390/genes12050679)
Supplement: Supplementary file 1 [file genes-12-00679-s001.zip › Supplementary materials/Table S1.docx]

Table S1. Disposition of patients by the type of treatment received besides immunotherapy

| **Characteristic** | **Immunotherapy**  **N = 15** | **No immunotherapy**  **N = 15** |
| --- | --- | --- |
| **Period of treatment** | After June 2015 | Before June 2015 |
| **Receipt of chemotherapy**  Yes  No | 11  4 | 15  0 |
| **Monochemotherapy** | 1**^*^** | 3**^**^** |
| **Platinum doublet** | 9 | 12 |
| **Type of platinum doublet**  Pemetrexed based  Non pemetrexed based | 5**^***^**  4 | 4  8 |
| **Line of platinum doublet**  First  Second or later | 5**^***^**  4 | 9  3 |
| **Line of EGFR-TKI treatment**  First  Second or later | 3  1 | 2  5 |
| **Type of EGFR-TKI treatment**  Gefitinib  Erlotinib  Afatinib  Osimertinib  Poziotinib | 1  1  1  -  1 | 1  5  -  1  - |

EGFR-TKI, epidermal growth factor receptor-tyrosine kinase inhibitor. **^*^**Vinorelbine;  **^**^**Gemcitabine;  ^*^**^**^**2 patients received chemotherapy with pembrolizumab
