# Supplementary figures and images for: Sensitivity to Immune Checkpoint Blockade in Advanced Non-Small Cell Lung Cancer Patients with EGFR Exon 20 Insertion Mutations
Source: Genes (Basel). 2021 Apr 30;12(5):679. doi: 10.3390/genes12050679 (PMC8147255; doi:10.3390/genes12050679)

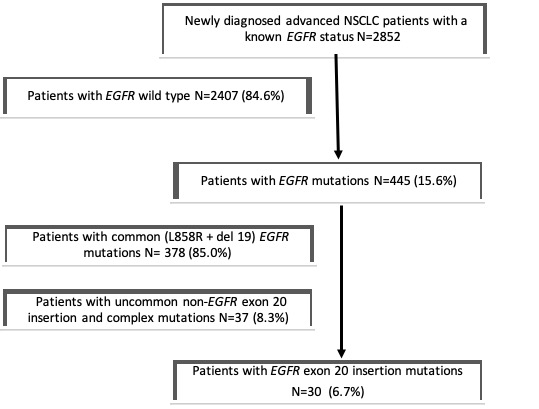

Supplement: Supplementary file 1 [file genes-12-00679-s001.zip › Supplementary materials/Figure S1.jpg]
